# A member of the CPW-WPC protein family is expressed in and localized to the surface of developing ookinetes

**DOI:** 10.1186/1475-2875-12-129

**Published:** 2013-04-15

**Authors:** Niwat Kangwanrangsan, Mayumi Tachibana, Rachaneeporn Jenwithisuk, Takafumi Tsuboi, Suda Riengrojpitak, Motomi Torii, Tomoko Ishino

**Affiliations:** 1 Department of Molecular Parasitology, Graduate School of Medicine, Ehime University, Shitsukawa, Toon, Matsuyama, Ehime, 791-0295, Japan; 2 Division of Molecular Parasitology, Proteo-Science Center, Ehime University, Shitsukawa, Toon, Matsuyama, Ehime, 791-0295, Japan; 3 Division of Malaria Research, Proteo-Science Center, Ehime University, Matsuyama, Ehime, 790-8577, Japan; 4 Department of Pathobiology, Faculty of Science, Mahidol University, Rachathevi, Bangkok, 10400, Thailand; 5 Present address: Department of Pathobiology, Faculty of Science, Mahidol University, Rachathevi, Bangkok, 10400, Thailand; 6 Present address: Mahidol Vivax Research Unit, Faculty of Tropical Medicine, Mahidol University, Rachathevi, Bangkok, 10400, Thailand

**Keywords:** Malaria, Transmission-blocking vaccine, Mosquito, Post-transcriptional regulation, CPW-WPC protein

## Abstract

**Background:**

Despite the development of malaria control programs, billions of people are still at risk for this infectious disease. Recently, the idea of the transmission-blocking vaccine, which works by interrupting the infection of mosquitoes by parasites, has gained attention as a promising strategy for malaria control and eradication. To date, a limited number of surface proteins have been identified in mosquito-stage parasites and investigated as potential targets for transmission-blocking vaccines. Therefore, for the development of effective transmission-blocking strategies in epidemic areas, it is necessary to identify novel zygote/ookinete surface proteins as candidate antigens.

**Methods:**

Since the expression of many zygote/ookinete proteins is regulated post-transcriptionally, proteins that are regulated by well-known translational mediators were focused. Through *in silico* screening, CPW-WPC family proteins were selected as potential zygote/ookinete surface proteins. All experiments were performed in the rodent malaria parasite, *Plasmodium yoelii* XNL. mRNA and protein expression profiles were examined by RT-PCR and western blotting, respectively, over the course of the life cycle of the malaria parasite. Protein function was also investigated by the generation of gene-disrupted transgenic parasites.

**Results:**

The CPW-WPC protein family, named after the unique WxC repeat domains, is highly conserved among *Plasmodium* species. It is revealed that CPW-WPC mRNA transcripts are transcribed in gametocytes, while CPW-WPC proteins are expressed in zygote/ookinete-stage parasites. Localization analysis reveals that one of the CPW-WPC family members, designated as PyCPW-WPC-1, is a novel zygote/ookinete stage-specific surface protein. Targeted disruption of the *pycpw-wpc-1* gene caused no obvious defects during ookinete and oocyst formation, suggesting that PyCPW-WPC-1 is not essential for mosquito-stage parasite development.

**Conclusions:**

It is demonstrated that PyCPW-WPC-1 can be classified as a novel, post-transcriptionally regulated zygote/ookinete surface protein. Additional studies are required to determine whether all CPW-WPC family members are also present on the ookinete surface and share similar biological roles during mosquito-stage parasite development. Further investigations of CPW-WPC family proteins may facilitate understanding of parasite biology in the mosquito stage and development of transmission-blocking vaccines.

## Background

Malaria is a serious tropical disease caused by infection with *Plasmodium* parasites that are transmitted via *Anopheles* mosquitoes. While intense efforts in malaria control have affected a gradual decrease in the global number of malaria cases and deaths every year since 2005, an estimated 3.3 billion people were still at risk of malaria infection in 2010 [1]. The World Health Organization (WHO) has recommended two major strategies currently in use for malaria control: the prevention of mosquito bites using long-lasting insecticide-treated mosquito nets (LLINs) and indoor residual spraying (IRS) and the management of malaria cases with artemisinin-based combination therapy (ACT) and rapid diagnosis [1,2]. Despite extensive attempts to develop vaccine strategies to inhibit parasite proliferation in human hosts (pre-erythrocytic- and erythrocytic-stage vaccines), no vaccines have been developed for clinical use to date [3]. In contrast, transmission-blocking vaccine (TBV) strategies, which aim to interrupt parasite development in the mosquito vector, have recently been revealed as effective methods for the elimination and eradication of malaria [4]. Moreover, it has been suggested that TBV, in combination with blood-stage vaccines or anti-malarial drugs, could prevent the emergence of vaccine- or drug-resistant parasites [5,6].

Malaria transmission to mosquito vectors is initiated when male and female gametocytes are acquired during blood sucking. Gametocytes undergo development into gametes in the midgut and then mate to form zygotes. Later, they mature into ookinetes, the invasive form of the parasite that can penetrate the midgut epithelium. The principle idea of TBV is to interrupt parasite mating, development, or invasion inside the mosquito midgut using antibodies produced in human hosts and acquired, together with gametocytes, by mosquitoes during the blood meal. Therefore, TBV has the potential to break the life cycle of parasites in mosquito vectors [7].

Screening for TBV target antigens typically focuses on proteins expressed on the surface of parasites during the sexual stages (gametocytes and gametes) and/or mosquito-stages (zygotes and ookinetes) [8,9]. Several surface proteins have been identified as candidates for TBV antigens, including P25, P28, P230, P48/45, and PCCps/LAPs (LCCL/lectin adhesive-like protein) [10,11]. Studies using membrane-feeding assays have demonstrated that anti-P25 and anti-P28 antibodies inhibit parasite invasion in the mosquito midgut [12]. However, stepwise clinical examinations, together with further screening for candidate antigens, are necessary for the development of TBV strategies.

To screen candidate antigens for TBV, two criteria were set: 1) proteins should be expressed exclusively from gametes to ookinetes, and 2) proteins should be localized to the surface of parasites. Progress in genetic manipulation has revealed a number of proteins that are important for parasite development in mosquitoes, such as calcium-dependent protein kinase 4 (CDPK4) in exflagellation [13], P48/45 and hapless 2/generative cell-specific 1 (HAP2/GCS1) in gamete fertilization [14-16], and several proteins in ookinete motility and invasion (reviewed in [17]). Most of these molecules are regulated post-transcriptionally by messenger ribonucleoproteins, development of zygote inhibited (DOZI), and homolog of worm CAR-I and fly Trailer Hitch (CITH) [18,19].

In the current study, a novel malaria protein family, CPW-WPC, which is expressed on the surface of developing ookinetes, is identified by focusing on predicted secretory and post-transcriptionally regulated proteins. These findings suggest that CPW-WPC family proteins may be potential candidate antigens for transmission-blocking strategies.

## Methods

### Experimental infection of mice by parasites

All the animal experiments were conducted in accordance with the guide for animal experimentation at Ehime University, Graduate School of Medicine. Eight-week-old female BALB/c mice (CLEA Japan Inc., Tokyo, Japan), pretreated with 0.2 mL of 0.6 mg/mL phenylhydrazine, were injected intraperitoneally with *P. yoelii* 17XNL parasites. To obtain only gametocytes, asexual-stage parasites were eliminated by intraperitoneal injection of 0.25 mL sulfadiazine (2 mg/mL) 24 h before blood collection.

### Collection of parasites

For blood-stage parasites, infected blood (at 10–20% parasitaemia) was mixed with 5 volumes of ice-cold incomplete medium (ICM; RPMI1640 medium, pH 7.4, Invitrogen) and then passed through a sterile CF11 filter (Whatman, England) to remove white blood cells. Cells were pelleted at 1,200 × *g* for 5 min and washed with ice-cold ICM before antigen preparation. For *in vitro* ookinete culture, infected blood was immediately mixed with 5 volumes of SA buffer (10 mM Tris, 150 mM NaCl, 10 mM glucose, pH 7.3, warmed to 37°C) to prevent gamete exflagellation and then passed through a sterile CF11 filter. After washing with ICM (warmed to 37°C), the parasites were resuspended with 20 volumes of ookinete culture medium (OCM; 24°C), composed of RPMI1640 medium containing 20% heat-inactivated fetal calf serum, 0.367 mM hypoxanthine, 25 mM 4-(2-hydroxyethyl)-1-piperazineethanesulfonic acid (HEPES), and 5 U/mL heparin (pH 8.3). The parasites were then transferred into culture flasks and incubated at 24°C. At 0, 1, 4, 16, and 24 h after incubation, equal volumes of cultured ookinetes were collected and washed with ice-cold ICM before antigen preparation.

### Preparation of parasite antigens

Parasite samples were purified by density gradient centrifugation using Percoll (GE Healthcare, USA). Briefly, parasite pellets were gently mixed with 4 volumes of ICM, layered onto 50% Percoll, and centrifuged at 410 × *g* for 20 min at room temperature. Purified parasites were collected from the interface, washed twice, and resuspended in 2 volumes of ICM. The purity was checked microscopically using Giemsa-stained blood smears, and the parasite number was determined by counting under a haemocytometer before spotting onto glass slides for the immunofluorescent assay. To prepare the antigens for western blotting, the purified parasites were treated for 10 min at 4°C with 0.15% saponin in ICM containing protease inhibitors (Roche, Germany). The samples were washed twice with 0.1 M phosphate-buffered saline (PBS) containing protease inhibitors (PBS-PI) and were stored as pellets at -80°C until use. For the collection of sporozoites, the salivary glands were dissected from infected mosquitoes at day 15 post-feeding and then ground in ice-cold ICM to release sporozoites.

### Bioinformatics

The genomic sequences used in this study were obtained from PlasmoDB, the Plasmodium Genomics Resource [20]. The putative signal peptide and CPW-WPC domains were also investigated. Multiple sequence alignments of CPW-WPC family members were performed with ClustalW (MegAlign; Lasergene®).

### RT-PCR of *pycpw-wpc-1* and CPW-WPC family member transcripts

Total RNA was isolated from *in vitro* cultured ookinetes using the RNeasy Micro Kit (Qiagen). To obtain cDNA, total RNA was subjected to reverse transcription and subsequent PCR using the PrimeScript reagent kit (TAKARA, Japan). The transcriptional profiles of *pycpw-wpc-1*, *py00599*, *py03515*, *py04297*, and *pyhsp70* were determined by conventional PCR at 28 cycles using gene-specific primers (see Additional file 1). cDNA concentrations were normalized across samples to *pyhsp70* expression. PCR products were analysed using ImageQuant LAS 4000 (GE Healthcare), and band intensities were measured using Adobe Photoshop (Adobe System Inc., USA).

### Expression of recombinant PyCPW-WPC-1 (rPyCPW-WPC-1)

The target gene was amplified from *P. yoelii* 17XNL zygote-enriched cDNA by PCR using the Phusion High-Fidelity DNA Polymerase (FINNZYMES, Finland). A gene-specific primer set (forward, 5^′^-CTCGAGAAAACGTTTGTCTTTTCTGGAGACG-3^′^ and –reverse, 5^′^-GGATCCTTAAATAATTGATCCAGTTATTGAATCC-3^′^) was designed to amplify the target sequence (1,539 base pairs), excluding the N-terminal signal sequence. The PCR fragment was cloned into the pEU-E01-GST-TEV-MCS-N2 vector (CellFree Sciences Co. Ltd., Matsuyama, Japan), as previously described [21]. Plasmids were then utilized for protein production in a wheat germ cell-free protein expression system using the bilayer translation reaction method [22,23]. GST-fused rPyCPW-WPC-1 was bound to a glutathione Sepharose 4B column, followed by cleavage with a tobacco etch virus protease to elute the rPyCPW-WPC-1 protein from the column. The yield was analyzed by Bradford’s assay and conventional SDS-PAGE with Coomassie brilliant blue staining.

### Production of anti-PyCPW-WPC-1 antibodies

Eight-week-old female BALB/c mice were immunized by intraperitoneal injection of rPyCPW-WPC-1 in Freund’s adjuvant (Wako, Japan) 3 times (30 μg in 0.2 mL each) at 3-week-intervals. Immune sera were obtained two weeks after the last immunization. The reactivity and specificity of anti-rPyCPW-WPC-1 antisera were determined by ELISA and western blotting.

### SDS-PAGE and western blotting

Parasite pellets were extracted with 1% Triton X-100 in PBS-PI. To solubilize the parasite antigens, the mixture was incubated on ice for 1 h. After centrifugation, the supernatant was recovered into a new microcentrifuge tube and mixed with loading buffer containing 2-β-mercaptoethanol at 4% of the final volume. Antigens were then loaded onto 12.5% polyacrylamide gels (ATTO Bioscience & Biotechnology, Japan) at 0.5–1 × 10^5^ parasites/lane and run in an electrophoresis buffer under a constant current. The separated proteins were then transferred onto PVDF membranes (Millipore) using a semidry transfer system. After transfer, membranes were incubated with 5% skim milk in PBS containing 0.1% Tween20 (PBST) at room temperature for 2 h, followed by incubation with mouse anti-PyCPW-WPC-1 antiserum (dilution, 1:100) for 1 h. After washing with PBST, the membranes were then incubated with horseradish peroxidase (HRP)-conjugated goat anti-mouse IgG (dilution, 1:20,000; Invitrogen) for 30 min. Unbound antibodies were washed out with PBST, and membranes were incubated with a chemiluminescent-HRP substrate (Immobilon Western, Millipore), wrapped in a cassette, and exposed to X-ray film (Fujifilm Corporation, Tokyo, Japan). Exposed films were developed using a CEPROS SV machine (Fujifilm Corporation).

### Indirect immunofluorescent assay (IFA)

Antigen slides were fixed with ice-cold acetone for 5 min. Nonspecific binding was then blocked with 5% skim milk in PBS at 37°C for 30 min before incubation with antibodies (mouse anti-PyCPW-WPC-1 at a dilution of 1:100 and rabbit anti-Pys25 at a dilution of 1:10,000) at 37°C for 1 h. The slides were then washed in PBS for 5 min and subsequently incubated with Alexa Fluor 488 goat anti-mouse IgG and Alexa Fluor 546 goat anti-rabbit IgG (Invitrogen) diluted in PBS containing 1 μg/mL 4^′^6-diamidino-2-phenylindole (DAPI) at 37°C for 30 min. After washing out the unbound antibodies, the coverslips were mounted with Prolong Gold antifade reagent (Invitrogen), and the slides were observed under a confocal microscope (LSM710; Carl Zeiss, Germany). The resulting images were analysed using the Zen software (Carl Zeiss, Germany).

### Targeted gene disruption of *pycpw-wpc-1*

*Pycpw-wpc-1* was disrupted by double-crossover homologous recombination in *P. yoelii* 17XNL. Two basic plasmids, pPbDT3UB12 and pHDEF1-mh-R12, were used for the construction of transfection plasmids, as previously described [24]. In brief, target sequences were amplified by PCR using KOD Plus DNA polymerase (Toyobo, Japan) with primer sets targeting the 809-base pair region upstream (-forward, 5^′^-CTCGAGTATGCATAATTGTGAATAGTTATTGG-3^′^ and –reverse, 5^′^-GGATCCGACCCCTATAATAATAAAAGGTCTGTC-3^′^) and 755-base pair region downstream (-forward, 5^′^-AGATCTACCAGATGATTATAATGGACCTTGTCC-3^′^ and –reverse, 5^′^-CTCGAGCGGATGTATTGAGAAGCTTTACATGTG-3^′^) of *pycpw-wpc-1*. PCR products were subsequently digested and inserted into pPbDT3UB12. Using Gateway Technology (Invitrogen), the modified pPbDT3UB12 plasmid was then subjected to a BP recombination reaction with the donor vector pDONR221 to give the entry plasmid. This entry plasmid was then subjected to an LR recombination reaction with pHDEF-1-mh-R12 to yield the final construct, which was linearized with *Xho*I before use. Enriched schizonts were transfected with 20 μg of linearized construct by electroporation using Nucleofector (Lonza) with a human T cell solution and the U-33 program. Transfected parasites were delivered into 8-week-old BALB/c female mice by intravenous injection. Twenty-four hours later, the mice were treated with pyrimethamine (70 μg/mL) via their drinking water. Infected blood was collected to examine the integration of the disruption cassette by PCR using the KOD FX DNA polymerase with a unique set of primers. Successfully transfected parasites were cloned using the limiting dilution technique, and cloned parasites were cultured for ookinetes. The expression of PyCPW-WPC-1 was measured by western blotting and IFA using mouse anti-PyCPW-WPC-1 antibodies.

### Mosquito feeding assay

More than 50 female *Anopheles stephensi* mosquitoes were allowed to fed on mice infected with wild-type- or gene-disrupted parasites, and only mosquitoes that showed complete engulfment of the blood were selected for an additional two weeks of incubation at 24°C. For infectivity analysis, mosquito midguts were dissected on day 10 post-feeding, and oocyst numbers were counted under a microscope. To examine sporozoite-formation ability, sporozoites were collected from midguts on day 15 post-feeding and counted.

## Results

### Identification of *Plasmodium* CPW-WPC family members

**Figure 1:**
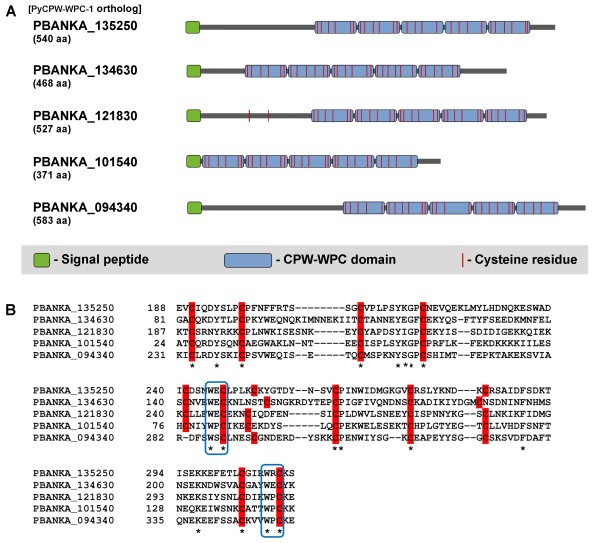
**Schematic representation of *Plasmodium *CPW-WPC family proteins.** (**A**) Diagram showing the common structure shared between CPW-WPC family proteins. Each protein contains a signal peptide and 5 repeated CPW-WPC domains, each having 4–6 cysteine residues. (**B**) ClustalW alignment of the first 2 CPW-WPC domains showing conserved amino acids (asterisks) and the arrangement of cysteines (highlight). Note that the WxC pattern is typically located at the end of each domain (box).

To identify novel mosquito-stage surface proteins, post-transcriptionally regulated proteins were explored since many zygote/ookinete proteins have been reported to be regulated post-transcriptionally. In a previous study, Mair *et al.* showed that the mRNA levels of 117 genes were dependent on both DOZI and CITH, which are well-studied messenger ribonucleoproteins expressed in gametocytes [19]. It was founded that seven of these 117 proteins belonged to the CPW-WPC family, which is named after the unique WxC motif found at the end of repeated domains. Amino acid sequence analyses showed that CPW-WPC family proteins are conserved among *Plasmodium* species and contain an N-terminal signal peptide. *In silico* analysis using PlasmoDB [25] demonstrated that eight CPW-WPC proteins share a similar structure, including a signal peptide and five repeated CPW-WPC domains with four to six cysteine residues (Figure 1A and Table 1). The sequence alignment of the first two domains is shown in Figure 1B. Moreover, CPW-WPC family proteins were also found in other members of *Apicomplexans*, such as *Theileria parva* (TP04_0816, TP04_0805, TP04_0558, and TP04_0183), *Babesia bovis* (BBOV_111009100, BBOV_111000280, BBOV_11004430, and BBOV_11004280), and *Toxoplasma gondii* (TGME49_066770, TGME49_047310, and TGME49_006440).

**Table 1:** Members of CPW-WPC domain protein family

**#**	**PBANKA (PY ortholog)**	**gDNA (bp)**	**coding seq. (bp)**	**amino acid (residues)**	**MW (kDa)**	**pI**	**SP**	**TM/GPI**	**CPW-WPC domain**	**DOZI KO vs. WT (confidence)**
1	135250 (PyCPW-WPC-1)	2,769	1,623	540	63.11	4.95	+	-	5	-2.71 (0.75)
2	134630 (PY04297)	1,407	1,407	468	55.01	7.04	+	-	5	-3.66 (0.72)
3	121830 (PY00599)	1,584	1,584	527	62.13	6.83	+	-	5	-2.22 (0.75)
4	101540 (PY00690)	2,843	1,116	371	42.99	6.69	+	-	5	-2.26 (0.73)
5	094340 (PY00905)	3,584	1,752	583	67.10	6.79	+	-	5	-2.06 (0.75)
6	144930 (PY03515)	1,786	1,644	547	63.33	8.48	-	-	5	-2.38 (0.73)
7	124520 (PY07114)	2,196	753	250	28.55	4.79	+	-	2	-2.46 (0.72)
8	112880 (PY04202)	1,958	840	279	32.29	4.31	+	-	2	-2.51 (0.72)
Data from PlasmoDB (http://plasmodb.org/plasmo)										

Here, one of the CPW-WPC family proteins (PBANKA_135250) was selected for further investigation in another rodent malaria parasite (*P. yoelii* 17XNL). Since the nucleotide sequence of the orthologous gene in *P. yoelii* (designated as PyCPW-WPC-1) has not yet been fully identified on PlasmoDB, PCR analyses of both genomic DNA and cDNA were performed to determine the primary sequence and its exon-intron structure. Multiple amino acid sequence alignment of the PyCPW-WPC-1 protein showed 93–94% and 50–55% identity among the orthologs related to rodent (PBANKA_135250 and PCHAS_135710) and human malaria parasites (PF3D7_1338800, PVX_082875, and PKH_121500), respectively. Furthermore, all eight CPW-WPC family members are described on PlasmoDB as DOZI-dependent post-transcriptionally regulated proteins. Taken together, these data suggest that CPW-WPC domain proteins tend to be expressed in zygotes and/or ookinetes.

### Expression of *pycpw-wpc-1* mRNAs before ookinete formation

**Figure 2:**
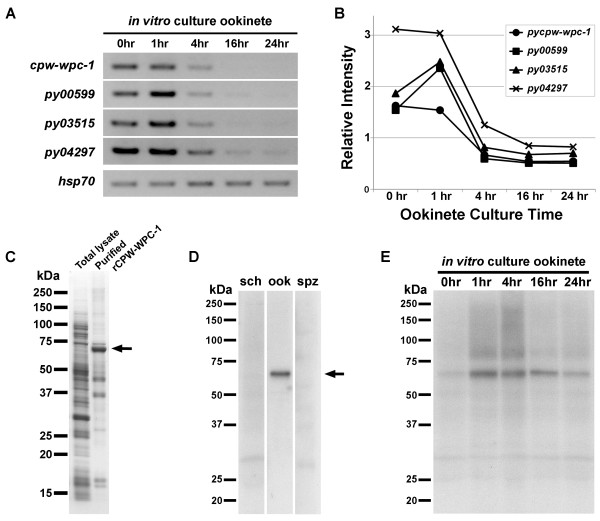
**Expression of PyCPW-WPC-1 mRNA and protein.** (**A**) mRNA expression profiles of *pycpw-wpc-1* and other CPW-WPC family members (*py00599*, *py03515*, and *py04297*). RT-PCR was performed using several time points for ookinete culture samples (0, 1, 4, 16, and 24 h). Template amounts were normalized between samples to the expression of *pyhsp70*, a housekeeping gene. (**B**) Quantitative analysis of *pycpw-wpc* family transcription. The bands intensities from Figure 2**A** were measured and plotted. (**C**) Recombinant PyCPW-WPC-1 (rPyCPW-WPC-1) synthesis using a wheat germ cell-free protein expression system. From the total lysate, GST-fused rPyCPW-WPC-1 was purified using a glutathione Sepharose column. Then, rPyCPW-WPC-1 was eluted from the column after cleavage by a tobacco etch virus protease. rPyCPW-WPC-1 was detected by SDS-PAGE at an approximate MW of 60 kDa (indicated by arrow). (**D**) PyCPW-WPC-1 protein expression profiles for all invasive stages. Expression of PyCPW-WPC-1 was examined in parasites at various invasive stages (schizont-enriched sample, sch; ookinete-enriched sample, ook; and salivary gland sporozoites, spz) by western blotting using anti-rPyCPW-WPC-1 antibodies. (**E**) PyCPW-WPC-1 protein expression during ookinete development. Specific bands corresponding to PyCPW-WPC-1 (indicated by arrow) were observed to be the strongest in 1- and 4-h ookinete culture samples, during which post-transcriptional regulation occurred (also see Figure 2**A**).

RT-PCR analyses were performed to investigate the expression profiles of *pycpw-wpc* family genes during mating and ookinete development *in vitro*. As shown in Figure 2A, *pycpw-wpc-1* transcripts were detected from 0–1 h of ookinete culture, which included gametocytes, gametes, and early zygotes. After zygote formation, the *pycpw-wpc-1* expression was reduced significantly (4 h) and was barely detectable after 16 and 24 h of ookinete culture (Figure 2A). Other family members (*py00599*, *py03515*, and *py04297*, corresponding to PBANKA_121830, PBANKA_144930, and PBANKA_134630, respectively) showed similar transcription patterns, i.e., highly expressed until zygote formation, with a subsequent decrease in expression. Among the examined molecules, *py04297*, which exhibited the highest expression, had the same expression pattern as *pycpw-wpc-1*, suggesting that these proteins may have coordinate functions (Figure 2B). In contrast, the expression of *py00599* and *py03515* peaked at 1 h of ookinete culture. These results reveal that *pycpw-wpc* family genes are expressed predominantly before zygote formation and that the expression of these targets decreases during the course of ookinete development.

### PyCPW-WPC-1 is a zygote/ookinete stage-specific protein

To obtain specific antibodies targeting PyCPW-WPC-1, full-length recombinant PyCPW-WPC-1 without a signal sequence (rPyCPW-WPC-1) was synthesized using a wheat germ cell-free protein expression system. After purification, rPyCPW-WPC-1 was obtained as the major band with a molecular weight of about 60 kDa (Figure 2C), and this purified product was then used for immunization in mice. Antisera were collected from immunized mice and used for western blotting to study the expression of the target protein during the malaria life cycle, including schizont-enriched blood-stage parasites, ookinetes, and sporozoites collected from mosquito salivary glands. The results demonstrated that PyCPW-WPC-1 was produced exclusively in ookinetes (Figure 2D). Moreover, western blotting analysis using the same *in vitro* cultured ookinete samples as used for transcriptional analysis (see Figure 2A) showed that PyCPW-WPC-1 expression was very low in gametes (0 h), despite the presence of *pycpw-wpc-1* transcripts. The expression of PyCPW-WPC-1 protein became prominent at 1 and 4 h of ookinete culture and decreased gradually until 24 h of ookinete maturation (Figure 2E). From morphological observations using Giemsa’s stain, it is concluded that translation of PyCPW-WPC-1 starts during zygote and early ookinete development. Together with our transcriptional data, these results demonstrate that the expression of PyCPW-WPC-1 is regulated post-transcriptionally, as predicted by microarray analyses of *pbdozi-*disrupted parasites [18].

### Localization of PyCPW-WPC-1 to the zygote/ookinete surface

**Figure 3:**
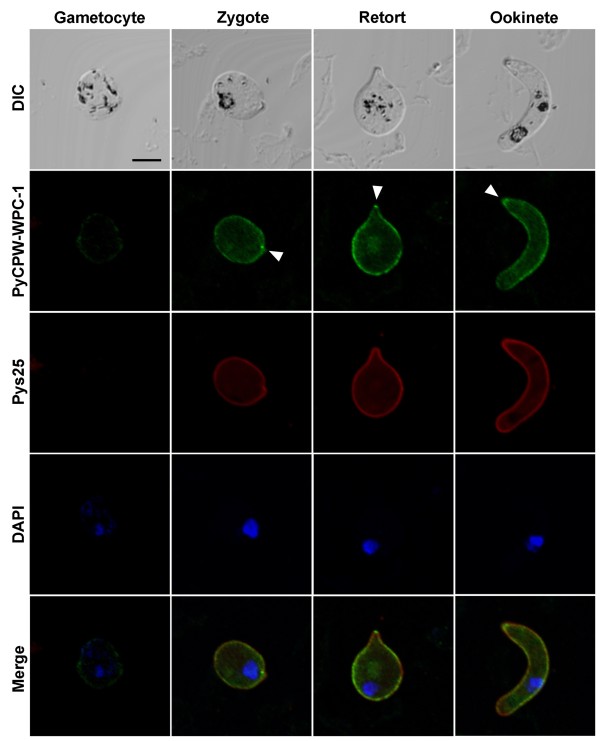
**Localization of PyCPW-WPC-1 in sexual-stage parasites.** Using an immunofluorescent assay (IFA), PyCPW-WPC-1 was found to be localized to the surface of developing ookinetes, including zygotes, retorts, and mature ookinetes (shown in green). The surface of developing ookinetes was stained with antibodies targeting Pys25 (red), a well-known ookinete surface protein. Nuclei were stained by DAPI (blue). The merged images represented the colocalization of PyCPW-WPC-1 and Pys25 on the parasite surface. Parasite morphology was analysed by differential interference contrast (DIC) imaging. The bar indicates 5 μm.

Next, the localization of PyCPW-WPC-1 during mosquito-stage parasite development, from gametocytes to ookinetes, was investigated. Immunofluorescent staining using mouse anti-PyCPW-WPC-1 antiserum revealed a strong signal on the surface of zygotes, retorts, and mature ookinetes, but not on gametocytes (Figure 3). The staining pattern was similar to that of Pys25, a well-known ookinete surface protein. In addition, another family member, Py03515, also showed a similar surface localization pattern (shown in Additional file 2).

### PyCPW-WPC-1 is not essential for ookinete formation

**Figure 4:**
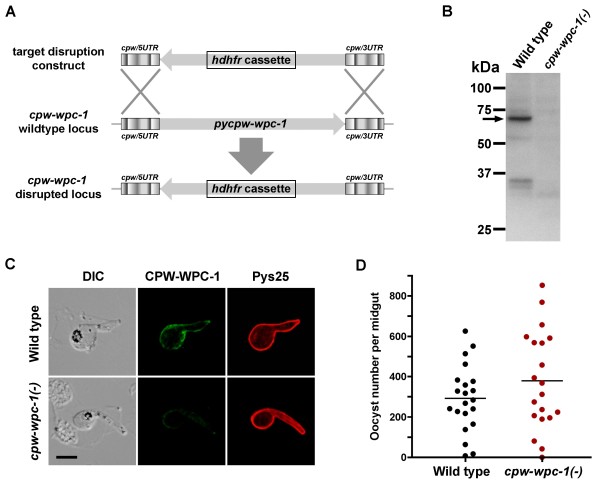
**Phenotypic analysis of *pycpw-wpc-1 *gene disruption in parasites.** (**A**) Schematic representation of *pycpw-wpc-1* gene disruption. To investigate the function of PyCPW-WPC-1 during sexual-stage development, the *pycpw-wpc-1* coding region was disrupted by insertion of the *hdhfr* cassette using double-crossover homologous recombination. (**B**) Depletion of PyCPW-WPC-1 protein in transgenic parasites. Ookinete antigens were prepared from *pycpw-wpc-1*-disrupted parasites and wild-type parasites. Western blotting using specific antibodies demonstrated the obvious reduction in PyCPW-WPC-1 protein expression as compared to the reference band from wild-type ookinete extracts (arrow). (**C**) Disappearance of the PyCPW-WPC-1 surface-localizing pattern in transgenic ookinetes. *Pycpw-wpc-1* gene-disrupted ookinetes appeared normal in morphology, as shown by DIC images (left panels) and surface localization of Pys25 (right panels). Compared to the wild-type parasite, *pycpw-wpc-1* gene-disrupted ookinetes exhibited little or no surface expression of PyCPW-WPC-1. The bar indicates 5 μm. (**D**) *pycpw-wpc-1*-disrupted parasites showed no defects in oocyst formation. The oocyst numbers were counted from individual midguts dissected from mosquitoes, which were fed on mice infected with wild-type or *pycpw-wpc-1*-disrupted parasites. Horizontal lines indicate mean oocyst numbers.

To investigate the function of PyCPW-WPC-1, targeted gene disruption by homologous recombination was performed (Figure 4A). Gene-disrupted parasites were selected by administration of antimalarial drugs to infected mice, and after cloning, disruption of the *pycpw-wpc-1* locus was confirmed by PCR using specific primers. To examine the formation of ookinetes by mutant parasites, *in vitro* ookinete cultures were grown. *pycpw-wpc-1*(-) ookinetes appeared to have a normal morphology (see Figure 4C), with an almost equivalent efficiency to the formation of wild-type ookinetes, indicating that PyCPW-WPC-1 is not essential for ookinete development *in vitro*. Using these cultured ookinetes, depletion of PyCPW-WPC-1 was confirmed by western blotting (Figure 4B) and immunofluorescent analyses (Figure 4C). The transmission ability of *pycpw-wpc-1*-disrupted parasites was analysed by mosquito feeding assay. As shown in Figure 4D, the oocyst number of *pycpw-wpc-1*(-) parasites was not significantly different from that of wild-type parasites examined by unpaired t-tests, indicating that CPW-WPC-1 is dispensable during oocyst formation *in vivo*. Furthermore, to examine the efficiency of sporozoites production, the numbers of sporozoites collected from midguts at day 15 post-feeding were compared. The average number of *pycpw-wpc-1*(-) sporozoites (19,783 ± 11,176) was equivalent to that of wild-type sporozoites (18,057 ± 4,977), strongly suggesting that CPW-WPC-1 is not essential for parasite transmission to mosquito vectors. On the other hand, since at least two members of the CPW-WPC protein family are co-expressed and colocalized on the ookinete surface, it is possible that other family members could compensate for the function of PyCPW-WPC-1 during ookinete development.

## Discussion

After the discovery that the zygote/ookinete surface proteins, P25 and P28, could be good candidate antigens for TBVs, considerable effort has been made to identify additional surface proteins; however, these attempts have not been successful. This study demonstrates that a member of the CPW-WPC protein family (PyCPW-WPC-1) is expressed during zygote and ookinete development in mosquitoes and is localized to the surface of developing ookinetes in *P. yoelii.* Database analyses using *P. berghei* demonstrate that the CPW-WPC family contains eight proteins, as shown in Table 1. Interestingly, studies have shown that the transcription of all CPW-WPC members is reduced in *pbdozi* mutants [18], indicating that all family members are expressed during the sexual stage under DOZI-dependent post-translational control. This characteristic can also be found in several hundred other proteins expressed in ookinetes, as demonstrated by studies on regulatory molecules, such as DOZI, CITH, HMGB2, and MISFIT [18,19,26,27].

Similar to the structure of P25 and P28, CPW-WPC proteins also contain conserved cysteine domains (Figure 1A). Despite the absence of transmembrane or glycosylphosphatidylinositol (GPI) anchor domains, two members of the CPW-WPC protein family were found to be localized to the ookinete surface (Figure 3). Moreover, by algorithmic analysis using MAAP [28], CPW-WPC proteins appeared in the predicted adhesin group, suggesting that they may be involved in host parasite interactions during ookinete development and/or migration in the mosquito midgut. It was also shown here that targeted *pycpw-wpc-1* gene disruption had no significant effect on ookinete or sporozoite formation. Since at least one other CPW-WPC protein (Py03515) showed a similar ookinete surface localization pattern (Additional file 2), it is possible that other family members could compensate for PyCPW-WPC-1 function. Therefore, it would be interesting to determine whether all CPW-WPC family members are expressed at the same time on the ookinete surface or whether there is exclusive expression of these proteins, as is seen for *var* genes, a family of *Plasmodium falciparum* virulence genes [29]. Thus, for further functional analysis of this protein family, double gene disruption should be attempted.

Due to their ookinete surface localization pattern, CPW-WPC family proteins may be potential candidates for transmission-blocking antigens, regardless of whether CPW-WPC-1 is dispensable in parasite transmission to mosquitoes. Previous reports have demonstrated that specific antibodies against P25 and P28 inhibited parasite transmission in mosquito midguts, although single disruption of each gene showed no clear defects during mosquito-stage development [12,30]. Moreover, CPW-WPC proteins are conserved among *Plasmodium* species, including *P. falciparum* and *Plasmodium vivax*, and CPW-WPC family proteins are less polymorphic than known blood-stage vaccine targets, such as apical membrane antigen 1 (AMA1) and merozoite surface protein 1 (MSP1). These characteristics are clinically important for vaccine development; therefore, further studies including transmission-blocking assays using specific antibodies against CPW-WPC family proteins should be required.

## Conclusions

The current study describes PyCPW-WPC-1, a novel post-transcriptionally regulated ookinete surface protein. This protein contains CPW-WPC domains, composed of conserved cysteine residues. *In silico* analysis shows that there are eight members of the CPW-WPC protein family, all of which are regulated by DOZI, a post-transcriptional regulatory factor that functions during parasite development in mosquitoes. By targeted gene disruption, it is demonstrated that PyCPW-WPC-1 is dispensable during ookinete development and invasion of midgut epithelium, which may suggest the redundancy of CPW-WPC family proteins. The identification of this novel ookinete surface protein family could be useful for understanding biology of mosquito-stage parasite development and for detecting possible candidates for transmission-blocking vaccine targets.

## Abbreviations

TBV: Transmission-blocking vaccine; DOZI: Development of zygote inhibited; CITH: Homolog of worm CAR-I and fly Trailer Hitch; ICM: Incomplete medium; OCM: Ookinete culture medium; HEPES: 4-(2-hydroxyethyl)-1-piperazineethanesulfonic acid; PBS: Phosphate-buffered saline; PI: Protease inhibitors; cDNA: Complementary DNA; PCR: Polymerase chain reaction; GST: Glutathione S transferase; SDS-PAGE: Sodium dodecyl sulfate-polyacrylamide gel electrophoresis; HRP: Horseradish peroxidase; IFA: Indirect immunofluorescent assay; DAPI: 4^′^6-diamidino-2-phenylindole

## Competing interests

The authors declare that they have no competing interests.

## Authors’ contributions

NK, TT, SR, MTR, and TI conceived, designed, and coordinated the study. NK, MTC, and RJ participated in the acquisition of data. NK, TT, SR, MTR, and TI analysed and interpreted the data. NK drafted the first version of the manuscript, and NK, MT, and TI were involved in critically revising the manuscript. All the authors have read and approved the final manuscript.

## Supplementary Material

Additional file 1 Gene-specific primers for transcriptional profiling. Click here for file

Additional file 2 Localization of PY03515 on the zygote/ookinete surface. Click here for file
