# Supplementary material for: A member of the CPW-WPC protein family is expressed in and localized to the surface of developing ookinetes
Source: Malar J. 2013 Apr 15;12:129. doi: 10.1186/1475-2875-12-129 (PMC3637178; doi:10.1186/1475-2875-12-129)
Supplement: Additional file 1 — Gene-specific primers for transcriptional profiling. [file 1475-2875-12-129-S1.pdf]

Additional file 1 - Gene-specific primers for transcriptional profiling.

genes	primer sets	PCR product size (bp)
pycpw-wpc-1	Fwd - 5′- GGATTCTAATAAAATAGAAGAAGT -3′<br>Rev - 5′- CCAACTTTCCTTCTGATTGTC -3′	199
py00599	Fwd - 5′- GGATAATTTTTAATGAAAATGAATG -3′<br>Rev - 5′- GCTTAGAGTTTCCTTCCATCC -3′	200
py03515	Fwd - 5′- GCTAAACAAATTTCTTTATCTACA -3′<br>Rev - 5′- CCTATATAATTTTTTGGAGCAATA -3′	203
py04297	Fwd - 5′- GGTCTTATGATTGTCCATATAAT -3′<br>Rev - 5′- GGTACAAATTTTTTATTTATACATG -3′	199
Py06158 (hsp70)	Fwd - 5′- GGTGTTAAAAGTTCATTAGAAGA -3′<br>Rev - 5′- CAGCATCTTGATATATTTTAGACA -3′	202
