# Supplementary figures and images for: A member of the CPW-WPC protein family is expressed in and localized to the surface of developing ookinetes
Source: Malar J. 2013 Apr 15;12:129. doi: 10.1186/1475-2875-12-129 (PMC3637178; doi:10.1186/1475-2875-12-129)

**Additional file 2 - Localization of PY03515 on the zygote/ookinete surface.**

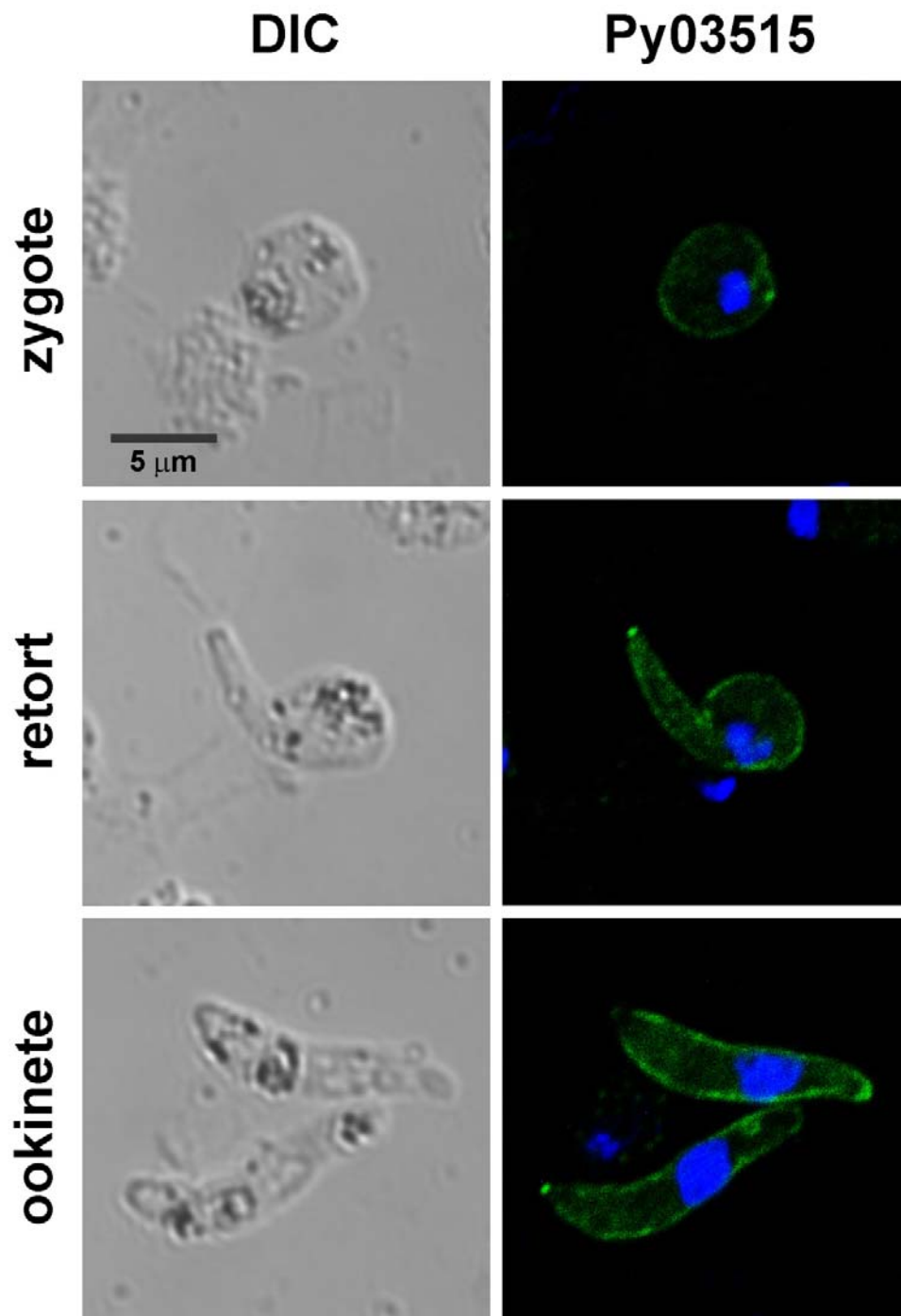

Supplement: Additional file 2 — Localization of PY03515 on the zygote/ookinete surface. [file 1475-2875-12-129-S2.pdf]
